# The Antibody Two-Step Solution

**DOI:** 10.12688/f1000research.7055.2

**Published:** 2015-12-22

**Authors:** Mike Browning

**Affiliations:** 1 Phosphosolutions LLC, Aurora, CO, 80045, USA

**Keywords:** Polyclonal, Monoclonal, Antibody, Variability, Validation, Pooled Serum

## Abstract

Problems with antibody quality have been described in numerous recent publications.  In the present commentary it is argued that these quality problems are due primarily to issues of antibody variability and antibody validation.  Further it is argued that the problem of antibody variability must be solved before validation can be useful.  A two-step solution to the antibody problem is thus proposed.

In the past year there have been a number of articles in Nature and other major journals that discuss the antibody problem in somewhat apocalyptic terms ^1^. In my mind there are only two main issues in the antibody problem: antibody validation, and antibody variability. Both of these issues have straightforward solutions that do not require any massive influx of cash or massive restructuring of antibody production.

Antibody validation is the hardest nut to crack and causes the most confusion. There is no consensus on what constitutes suitable validation and this is complicated by the different methods for antibody use. However, antibody validation is a process like all science where knowledge increases as more and more work is done with the antibody. Many journals now insist that antibody validation data be provided and this is a key part of the solution. As long as the data are clear and the methods used adequately described, progress in validation will occur with time. However, none of this progress will matter unless we deal with the antibody variability problem. What difference does it make if an antibody is validated if it is not possible to obtain the same antibody for future work?

There are two main reasons for the variability in an antibody’s performance. The first is that once an antibody is found to have a high demand, many different antibody manufacturers will try to make their own version of the antibody so they can sell it. But all these new antibodies will differ in unknown and unpredictable ways from the original antibody. Thus validation done on the original antibody may or may not be true for the new antibodies. One way to deal with this problem was recently suggested by Andrew Chalmers and his colleagues ^2^. They argue that all publications using commercial antibodies should report the name of the supplier and the catalog number of the antibody used. That way even if a supplier sells many varieties of the antibody a researcher will be able to order the same antibody that was used in the publication. Subsequently Bandrowski *et al.* ^3^ proposed an even more detailed and efficient antibody identification protocol with their Research Resource Identifiers (RRIDs) which are based on accession numbers assigned by an authoritative database. These suggestions are being incorporated into the instructions to authors in more and more journals.

Even though this action would greatly improve the value of antibody validation, an additional source of antibody variability would remain, namely lot-to-lot variability. This variability occurs because even if one buys the same antibody with the same catalog or RRID number, one still often encounters large variability in different lots of the same antibody obtained from different bleeds of the same animal or bleeds from different animals. There is a very straightforward fix to this type of variability. The solution is to pool all the positively screened serum collected from the animals. Virtually all lot-to-lot variability can be eliminated for polyclonal antibodies if this procedure is used. The antibody manufacturer could simply label the antibody as “pooled serum” to denote this fact. If this procedure is followed, it will no longer be necessary to reinvent the antibody validation wheel each time an antibody is used. Thus science can build upon itself as it is supposed to do.

Some may argue that one should use monoclonal antibodies to eliminate variability. This is unnecessary and also unwise. It is unnecessary because for most antibodies a single rabbit can produce a stable 20–30 year supply of antibody. Only a small percentage of all antibodies sold ever sell more than can be produced by a single rabbit. It is unwise because monoclonals cost at least 3X what polyclonals cost and we are unlikely to see a time in the near future when cost will be irrelevant. Moreover, polyclonal antibodies have been shown to be superior to monoclonal antibodies in a number of different applications ^4^.

Scientists and journals can fix the validation problem if antibody manufacturers will first fix the variability problem. This is called the Antibody Two-Step Solution.

## References

[1] Baker M: Reproducibility crisis: Blame it on the antibodies. *Nature.* 2015;521(7552):274–276. 10.1038/521274a 25993940

[2] Helsby MA Fenn JR Chalmers AD: Reporting research antibody use: how to increase experimental reproducibility. *F1000Res.* 2013;2:153. 10.12688/f1000research.2-153.v2 24358895 PMC3829129

[3] Bandrowski A Brush M Grethe JS: The Resource Identification Initiative: A cultural shift in publishing [version 2; referees: 2 approved]. *F1000Research.* 2015;4:134. 10.12688/f1000research.6555.2 26594330 PMC4648211

[4] Lipman NS Jackson LR Trudel LJ: Monoclonal versus polyclonal antibodies: distinguishing characteristics, applications, and information resources. *ILAR J.* 2005;46(3):258–268. 10.1093/ilar.46.3.258 15953833

