# Supplementary material for: The Antibody Two-Step Solution
Source: F1000Res. 2015 Dec 22;4:810. Originally published 2015 Sep 15. [Version 2] doi: 10.12688/f1000research.7055.2 (PMC4706045; doi:10.12688/f1000research.7055.2)
Supplement: Peer review history

# Peer review: Referee response for version 1

**DOI:** 10.5256/f1000research.7595.r10697

**Published:** 2015-10-06

**Authors:** Angus Nairn

**Affiliations:** 1 Department of Psychiatry, Yale School of Medicine, New Haven, CT, USA

This short commentary article presents an opinion on the ongoing discussion of quality control of commercial antibodies. This is an important topic given the importance of these reagents to progress in a wide range of biomedicine, and of the large economic costs involved in their successful use. But more importantly of the costs when they fail, are subject to variable success, or when they introduce error and artifact.

The comments come from the CEO of PhosphoSolutions, one of many companies who provide and generate general and state-specific antibodies to a wide variety of proteins and epitopes, and who have a vested interest in providing a high quality product. Two general issues are discussed, namely antibody variability and antibody validation. These are closely interconnected aspects of quality control, and two reasonable solutions are presented. In terms of variability, which arises for a number of different reasons, including batch-to-batch differences in a single source reagent, to multiple variants of protein or epitope-specific reagents generated by different individuals or companies, the suggestion to provide a catalog number and supplier is a reasonable start. These sorts of detail should be a minimal requirement, and have been discussed in other recent initiatives (see for example Helsby et al cited; Bandrowski et al http://dx.doi.org/10.12688/f1000research.6555.1).

But why not be more specific and provide full details of the characterization of the antigen, epitope if known, and precise lineage related to the origins of the reagent. Importantly, there needs to be specific information that indicates which particular batch is being sold. One could even consider some sort of unique bar code that would enable investigators to keep track electronically of the reagent they receive. Greater transparency and greater detail would benefit all parties involved.

In terms of variability, the reasonable suggestion is made to pool batches to avoid batch to batch variation. The context of the suggestion is that the discussion here is most relevant to the generation of rabbit polyclonal antisera and it makes sense. But in logistical terms, typically initial studies of a particular rabbit serum is done on a pilot basis to establish quality, and antibodies are often affinity purified in batches. Decision points may vary depending on whether the best strategy is to pool sera, or pool batches of purified antibody. A key missing element is the establishment of robust criteria to establish a quantitative threshold of an acceptable quality that will consistently ensure success of whatever pools of serum or purified antibody are used eventually in the desired application, be it immunoblotting, immunohistochemistry or ELISA.    

A third short ending deals with the strengths and weaknesses of monoclonal versus polyclonal antibodies. This is really a different issue from the rest of the discussion of validation and variability which is most relevant to rabbit polyclonal antiserum/antibodies. I suggest that the material in the last paragraph be used as part of a slightly revised introduction and used to set up the context for the later discussion. Monoclonal antibodies clearly have the capacity to overcome the variability issue and are more routine to validate once an assay is standardized. The second sentence “This is unnecessary and unwise” would be best removed, and the strengths of polyclonal antibodies (price and amount per rabbit, superior use in different applications) still made.

Another suggestion would be to remove the ambiguous comparison that “validation is the hardest nut to crack” while also saying “the variability problem” is most important. Better to say that variability and validation are two inter-connected issues, both of which need to have improved quality control.

I have read this submission. I believe that I have an appropriate level of expertise to confirm that it is of an acceptable scientific standard.

# Peer review: Referee response for version 1

**DOI:** 10.5256/f1000research.7595.r10334

**Published:** 2015-09-29

**Authors:** Andrew Chalmers

**Affiliations:** 1 Department of Biology and Biochemistry, University of Bath, Bath, UK

The opinion piece by Mike Browning makes a clear proposal to reduce the problems associated with using research antibodies. He proposes a two-step process, where step 1 is to reduce variability and the second step is to improve validation. The main contribution seems to me to be highlighting the importance of variability- step 1.

Overall it is nicely written, concise and makes a very useful contribution to the ongoing discussion about how to reduce the problems with research antibodies, something which is crucial to support progress and reproducibility in life sciences. I feel it is suitable for indexation.

I had a couple of comments/questions about the commentary:

1. A reasonable conclusion from the argument about variability seems to me that we should be working towards encouraging researchers to record and report lot numbers of the antibodies they use. Do you feel this would be beneficial?
2. The suggestion of pooling positive serum seems very sensible. I would be interested to know how frequently is this done and if it is not done very often why is this?

I have read this submission. I believe that I have an appropriate level of expertise to confirm that it is of an acceptable scientific standard.

# Peer review: Referee response for version 1

**DOI:** 10.5256/f1000research.7595.r10333

**Published:** 2015-09-25

**Authors:** C. Fernando Valenzuela

**Affiliations:** 1 Department of Neurosciences, School of Medicine, UNM Health Sciences Center, University of New Mexico, Albuquerque, NM, USA

The paper of Browning proposes a two-step solution to solve problems related to antibody performance in biomedical research. The author proposes that antibody variability must be fixed first (for instance, by pooling positive serum from multiple animals). Once this issue has been addressed, validation will become more straightforward and will progress with time. This well-written opinion article is timely and addresses an important issue that has been the focus of intense discussion in recent years.  There are only a few issues for the author’s consideration:

 

1. Although it is clear what the author means by the two-step solution, relatively little detail is given regarding the issue antibody validation.  A more detailed discussion of this issue is warranted.  For instance, what is the author’s take on the approach proposed by Bordeaux *et al. *(Biotechniques. 2010 Mar;48(3):197-209. doi: 10.2144/000113382). It is stated that antibody validation will essentially be accomplished as more and more work is done with the antibody.  However, there are many factors that limit the access of this information to both suppliers and users.  If the antibody does not work on a particular application, the researcher often discards it without reporting any problems in any form.  Perhaps being more aggressive about requesting feedback and making sure that all validation information is included in technical data sheets could contribute to solve the problem; the quality of the information included in some web sites and technical sheets is not particularly impressive.  Encouraging users to submit information to sites such as antibodypedia.com or histoneantibodies.com could also be a step forward. Several companies provide trial size antibodies that allow the users to test them in a particular application in exchange for feedback.
2. What are the author’s thoughts on how the task of antibody validation be distributed between vendors and users?
3. Regarding reporting of antibody use, in addition to reporting catalog numbers, it seems as if it would be crucial to report lot numbers as well.
4. What is the author’s take on the proposal that an independent body be recruited (e.g., National Institute of Standards) to oversee an antibody certification program?
5. The abstract does not explicitly define the two-step solution.

I have read this submission. I believe that I have an appropriate level of expertise to confirm that it is of an acceptable scientific standard.

# Response

**Published:** 2015-10-12

**Authors:** Mike Browning

**Affiliations:** PhosphoSolutions LLC, USA

Many thanks for your comments Dr. Valenzuela. I share your frustration with the issue of antibody validation. However, if variability is not dealt with first, validation has little value to subsequent studies. Moreover the issue is very complex and beyond the scope of this opinion article. I have discussed my perspectives of this issue in my blog. You offer a number of suggestions about validation that I strongly support. These include the proposal you cite by Bordeaux *et al*., the need for publishing negative data and contribution to sites that catalog validation data.

One issue you raise that I’d like to expand on is the use of lot numbers. Lot numbers can be both a blessing and a curse. Unfortunately, the use of lot numbers sometimes masks a tendency among suppliers to fail to follow best practices that will eliminate most antibody variability. The pooling of serum is the first best practice that must be followed to eliminate variability. Unfortunately very few suppliers do this. This is due primarily to an increase in cost and effort needed for pooling serum and also possibly to ignorance of best practices among some suppliers. The increase in cost is due to the fact that sales of the antibody must be delayed until all bleeds of the rabbits have been collected. The increase in effort is due to the need to screen all bleeds to determine which bleeds should be pooled. Many suppliers start selling an antibody as soon as they get a positive bleed. Then when that bleed is exhausted they switch to the next etc. However there can be huge differences in the quality of the antibody in subsequent bleeds. Serum pooling must be used to avoid this form of lot to lot variability.

There is a second even more serious form of antibody variability that is often obscured by simply changing the lot number. This results when an antibody supplier runs out of all the bleeds from the original animal. Some antibody suppliers then use new animals to make the antibody and simply denote this fact by changing the lot number. The potential differences between bleeds from the same animal pale beside the differences seen in different animals. It is not enough to simply change the lot number. The best practice is to modify the catalog number to reflect to new animal source.

Lastly, with regard to antibody certification by some independent body we wholeheartedly support such efforts to develop a set of best practices. One key issue in such an effort would need to be communication between all the stakeholders including researchers, the journals AND the suppliers.
